# Silica nanoparticle-exposure during neuronal differentiation modulates dopaminergic and cholinergic phenotypes in SH-SY5Y cells

**DOI:** 10.1186/s12951-019-0482-2

**Published:** 2019-04-01

**Authors:** Linda Wiedmer, Angélique D. Ducray, Martin Frenz, Michael H. Stoffel, Hans-Rudolf Widmer, Meike Mevissen

**Affiliations:** 1 Division of Veterinary Pharmacology and Toxicology, Vetsuisse Faculty, University of Bern, Laenggassstrasse 124, 3012 Bern, Switzerland; 2 Institute of Applied Physics, University of Bern, Bern, Switzerland; 3 Division of Veterinary Anatomy, Vetsuisse Faculty, University of Bern, Bern, Switzerland; 4 Department of Neurosurgery, Research Unit, Inselspital, University of Bern, Bern, Switzerland

**Keywords:** Neuronal differentiation, Phenotype, Nanomedicine, Toxicity, Polymeric-nanoparticles

## Abstract

**Background:**

Silica-ε-polycaprolactone-nanoparticles (SiPCL-NPs) represent a promising tool for laser-tissue soldering in the brain. After release of the SiPCL-NPs in the brain, neuronal differentiation might be modulated. The present study was performed to determine effects of SiPCL-NP-exposure at different stages of neuronal differentiation in neuron-like SH-SY5Y cells. The resulting phenotypes were analyzed quantitatively and signaling pathways involved in neuronal differentiation and degeneration were studied. SH-SY5Y cells were differentiated with *all*-*trans* retinoic acid or staurosporine to obtain predominantly cholinergic or dopaminergic neurons. The resulting phenotype was analyzed at the end of differentiation with and without the SiPCL-NPs given at various times during differentiation.

**Results:**

Exposure to SiPCL-NPs before and during differentiation led to a decreased cell viability of SH-SY5Y cells depending on the differentiation protocol used. SiPCL-NPs co-localized with the neuronal marker β-3-tubulin but did not alter the morphology of these cells. A significant decrease in the number of tyrosine hydroxylase (TH) immunoreactive neurons was found in staurosporine-differentiated cells when SiPCL-NPs were added at the end of the differentiation. TH-protein expression was also significantly downregulated when SiPCL-NPs were applied in the middle of differentiation. Protein expression of the marker for the dopamine active transporter (DAT) was not affected by SiPCL-NPs. SiPCL-NP-exposure predominantly decreased the expression of the high-affinity choline transporter 1 (CHT1) when the NPs were given before the differentiation. Pathways involved in neuronal differentiation, namely Akt, MAP-K, MAP-2 and the neurodegeneration-related markers β-catenin and GSK-3β were not altered by NP-exposure.

**Conclusions:**

The decrease in the number of dopaminergic and cholinergic cells may implicate neuronal dysfunction, but the data do not provide evidence that pathways relevant for differentiation and related to neurodegeneration are impaired.

## Background

Nanomaterials are used in medical applications including drug delivery in the central nervous system (CNS) [1]. Recently, biodegradable scaffolds with silica-ε-polycaprolactone-nanoparticles (SiPCL-NPs), bovine serum albumin (BSA) and indocyanine green have been developed for suture-less tissue fusion, namely laser-tissue soldering (LTS). LTS provides a promising alternative treatment method for injuries of blood vessels, offering shorter procedure time, immediate water tightness, faster wound healing and reduced recovery time as compared to classical microsuturing [2–4]. This technique makes use of a degradable polymer scaffold containing albumin and the chromophore indocyanine green (ICG). Tissue fusion is achieved by laser irradiation that results in denaturation of the BSA [5–7]. The breakdown of biodegradable implant leads to NP-release into the surrounding tissue. Therefore, interactions of nanomaterials with neurons and other cells in the brain need to be studied to estimate the possible health hazards. Intranasal administration of silica nanoparticles (Si-NPs) in rats was reported to induce oxidative stress and apoptosis in the striatum [8] and increase apoptotic cell death in hypothalamic neuronal cells (GT1) [9]. Similarly, oxidative damage and increased inflammation were found in the striatum of rats after intranasal application of Si-NPs. In vitro studies revealed a decrease in cell viability, an induction of oxidative stress, apoptosis and a depletion of dopamine accompanied by a decrease of tyrosine hydroxylase (TH) in the striatum as well as a in PC12 cells [10]. On the other hand, although oral administration of Si-NPs in *Drosophila melanogaster* resulted in an uptake into larval and adult neuronal tissues, neuronal cell viability was not affected [11]. Uptake of SiPCL-NPs designed for laser-tissue soldering [5, 6] has been demonstrated for microglia and neuron-like SH-SY5Y cells. SiPCL-NPs did not affect cell viability, cytotoxicity and apoptosis but led to a depletion of glutathione indicating oxidative stress [12]. The same NPs did not induce inflammation and autophagy in microglial cells [13] but impaired mitochondrial function in SH-SY5Y cells [14]. Notably, Si-NPs were shown to increase the production of reactive oxygen species and reactive nitrogen species in primary microglial cells [15], similarly to effects shown with silver nanoparticles (AgNPs) [16]. As mitochondrial dysfunction and oxidative stress have been demonstrated to play an important role in the development of neurodegenerative diseases [17, 18], and if NPs compromise neuronal differentiation and related signaling pathways [19–21], they pose a risk for neurodegeneration. Dayem et al. [22] demonstrated an increase in neurite length and an enhanced expression of neuronal differentiation markers after AgNP-exposure in SH-SY5Y cells. In contrast, neurite outgrowth was not modulated by SiPCL-NP-exposure in SH-SY5Y cells but led to a reduction of neuronal differentiation [23]. Zinc oxide NPs (ZnONPs) were reported to induce MAP-K/ERK phosphorylation in primary astrocytes [24]. In contrast, SiPCL-NP were shown to moderately decrease phosphorylated MAP-K in neuron-like SH-SY5Y cells [23]. Qiao et al. demonstrated an inhibition of PI3K/Akt, a pathway known to be involved in neuronal differentiation [14], neuronal survival [25] and neurogenesis [26], leading to a complete inhibition of neuronal differentiation [27]. In PC12 cells, SiNPs were shown to suppress phosphorylation of PI3K and Akt [28]. On the other hand, AgNP- and SiPCL-NP-exposure were demonstrated to upregulate phosphorylated Akt in SH-SY5Y cells [14, 22]. The Wnt/β-catenin-pathway is involved in the development and maintenance of the nervous system [29]. Activation of this pathway was reported to prevent neuronal death [17, 30], while a decrease in Wnt-signaling is related to the pathogenesis of neurodegeneration [31, 32]. In line with this notion, titanium dioxide NPs (TiO_2_NPs) have been demonstrated to significantly decrease the expression of markers of the Wnt-pathway [33].

Alterations of the dopaminergic phenotype may pose a threat towards neurodegeneration especially in Parkinson’s disease [34]. As cells undergo complex morphological, biochemical and functional shifts [35], NP-exposure needs to be studied at various times during differentiation. In this study, neuronal differentiation, consequential cellular phenotypes and the underlying signaling pathways [17, 26, 31] were investigated after SiPCL-NP-exposure using various differentiation protocols.

## Results

### Viability of differentiated SH-SY5Y cells after NP-administration

**Fig. 1:**
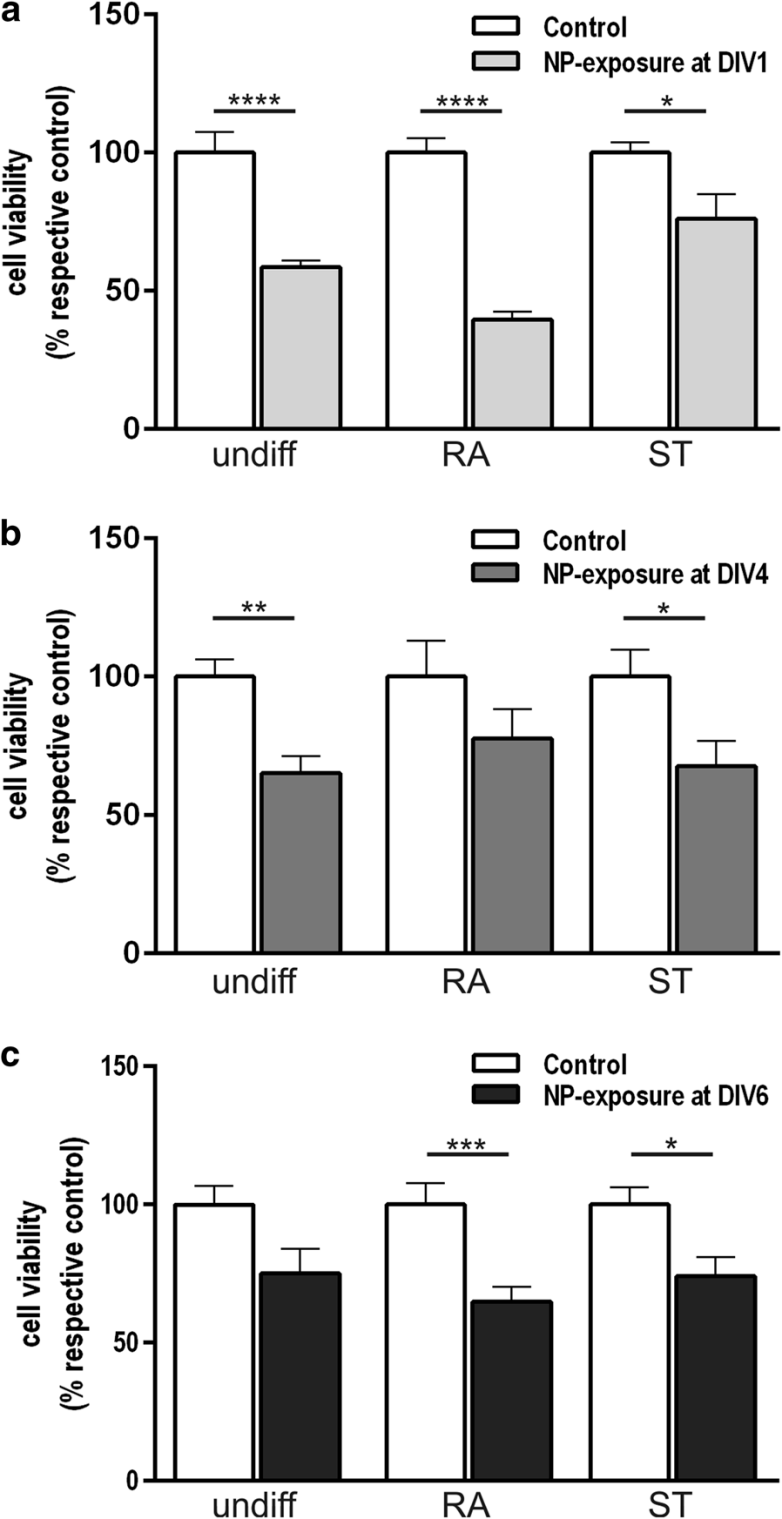
SH-SY5Y cells were differentiated with retinoic acid (RA) or staurosporine (ST). Undifferentiated cells were grown in parallel. SiPCL-NP-exposure was performed at a concentration of (2.6 × 10^10^ NPs/ml) for 24 h at day in vitro (DIV) 1 (**a**), DIV4 (**b**) or DIV6 (**c**). Cell viability was analyzed on DIV7 in undifferentiated and differentiated cells. The data represents the mean percentage (%) of the cell viability in relation to the respective controls. Error bars correspond to SEM. Significant differences between control- and NP-exposed-groups are labeled with asterisks (*p ≤ 0.05; **p ≤ 0.01; ***p ≤ 0.001; ****p ≤ 0.0001)

Effects of SiPCL-NPs designed for LTS in the brain [5–7, 36] were studied in SH-SY5Y cells during neuronal differentiation. SiPCL-NPs at a concentration of 2.6 × 10^10^ NPs/ml (24.9 μg/ml) significantly decreased the cell viability in all-trans retinoic acid (RA)- and staurosporine (ST)-differentiated SH-SY5Y cells with the effect depending on the specific timing of the exposure and the differentiation-supplement used (Fig. 1a–c). Cell viability was significantly reduced after SiPCL-NP-incubation at day in vitro (DIV) 1 in undifferentiated, RA-differentiated and ST-treated cells, respectively with the effect being more pronounced in RA-treated cells (Fig. 1a). SiPCL-NP-administration at DIV4 diminished the viability of ST-differentiated cells significantly, whereas no reduction was found in RA-treated cells. NP-exposure significantly reduced the cell viability in undifferentiated cells when given at DIV4 but not at DIV6 (Fig. 1b–c). Significant changes in cell viability were found when SiPCL-NPs were exposed at DIV6 regardless of the differentiation supplement used (Fig. 1c).

### Morphology of differentiated SH-SY5Y cells

**Fig. 2:**
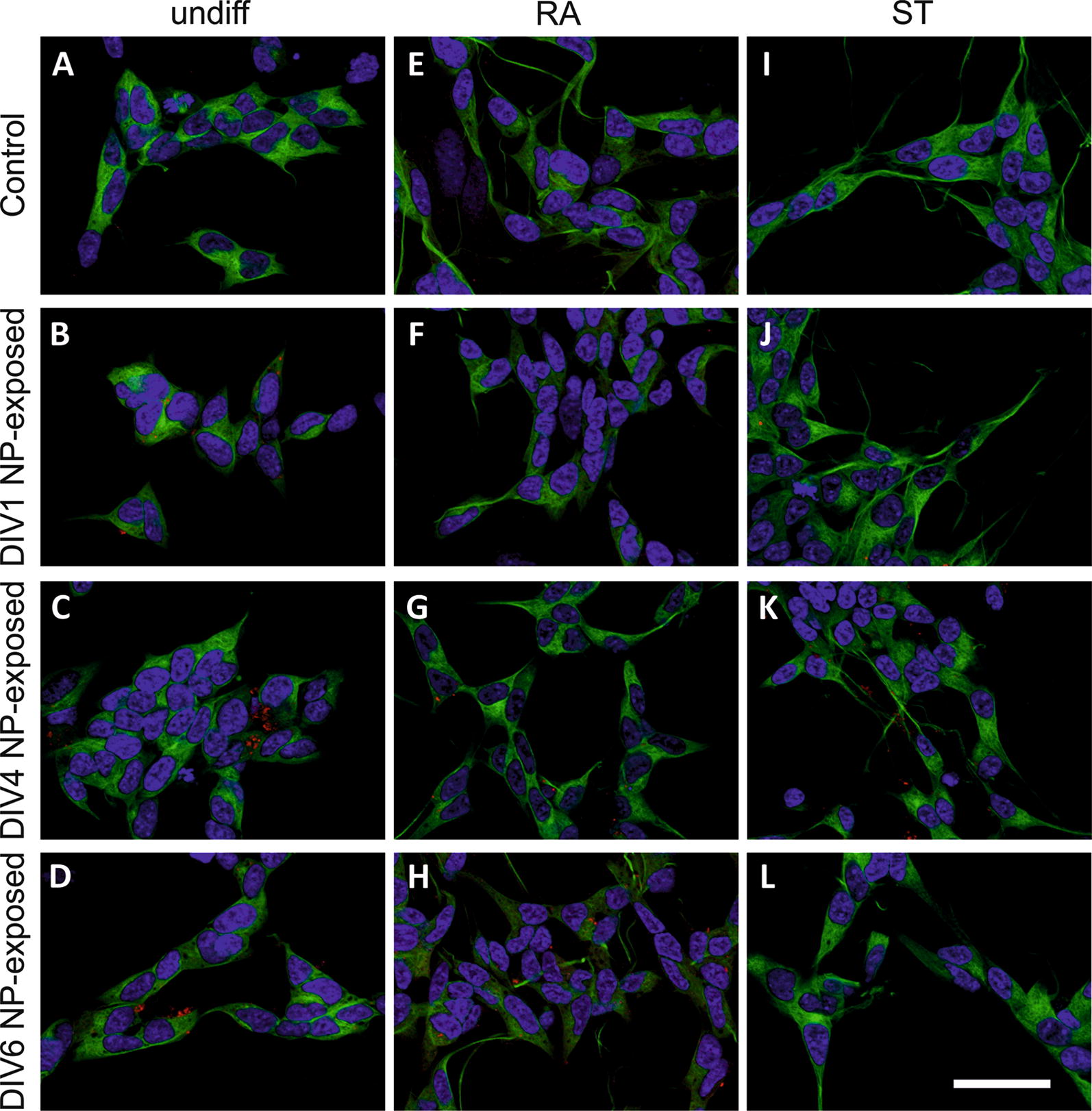
Representative immunofluorescence microscopy images showing the morphology of SH-SY5Y cells on day in vitro (DIV) 7, stained with b-3-tubulin (green). Cells were kept undifferentiated (**A**–**D**), differentiated with retinoic acid (RA) (**E**–**H**) or staurosporine (ST) (**I**–**L**). Control images, unexposed to NPs, are shown in A, B and C. SH-SY5Y cells exposed to (2.6 × 10^10^ NPs/ml) SiPCL-NPs (red) for 24 h at DIV1 are depicted in **B**, **F** and **J**. SH-SY5Y cells exposed to NPs at DIV4 are shown in **C**, **G** and **K** and exposure at DIV6 in **D**, **H** and **L**. Nuclei are shown in blue. Magnification ×630, scale bar = 50 μm

Naïve undifferentiated SH-SY5Y cells grew in clusters and the cell bodies appeared globular with only short neuronal processes in control cells (Fig. 2A) as well as after SiPCL-NP-exposure at DIV1, DIV4 and DIV6 (Fig. 2B–D). RA- (Fig. 2E–H) and ST-differentiated (Fig. 2I–L) SH-SY5Y cells showed longer processes that interconnected and formed a network. Incubation with rhodamine-labeled SiPCL-NPs showed that the NPs were located close to the nuclei, and they co-localized with the neuronal marker β-3-tubulin. NPs did not cause any obvious changes in cell morphology (Fig. 2B–D, F–H, J–L) or the expression of α/β-synuclein (data not shown).

### Quantitative analyses of the cellular phenotype

**Fig. 3:**
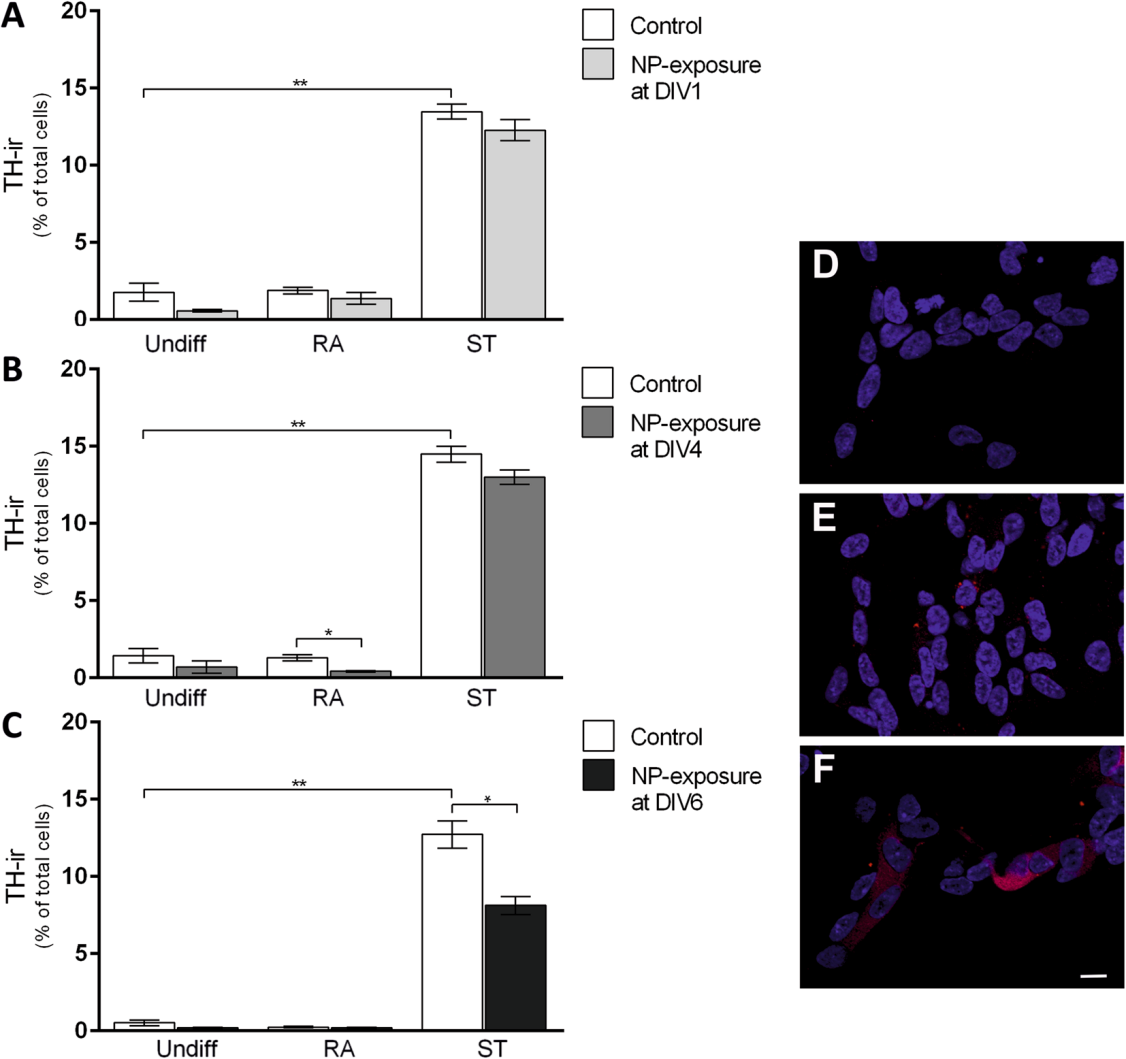
The effect of SiPCL-NP-exposure on tyrosine hydroxylase-immunoreactive (TH-ir) cell numbers was studied in SH-SY5Y cells undifferentiated (Undiff) or differentiated with retinoic acid (RA) or staurosporine (ST). SiPCL-NPs were incubated for 24 h at day in vitro (DIV) 1 (**A**), DIV4 (**B**) or DIV6 (**C**) at a concentration of (2.6 × 10^10^ NPs/ml). The dopaminergic phenotype was analyzed at DIV7. The data represent TH-ir cells and are given as mean percentage (%) of TH-ir cells of the total number of cells. Error bars correspond to SEM. Significant differences between control- and NP-exposed-groups are labeled with asterisks (*p ≤ 0.001; **p ≤ 0.0001). Representative immunofluorescence microscopy images of TH-ir cells (red) are depicted in **D**–**F** (**D** = Undiff NP DIV4, **E** = RA NP DIV4, **F** = ST NP DIV4). NPs are shown in red and cell nuclei in blue. Magnification ×630, scale bar = 10 μm

**Fig. 4:**
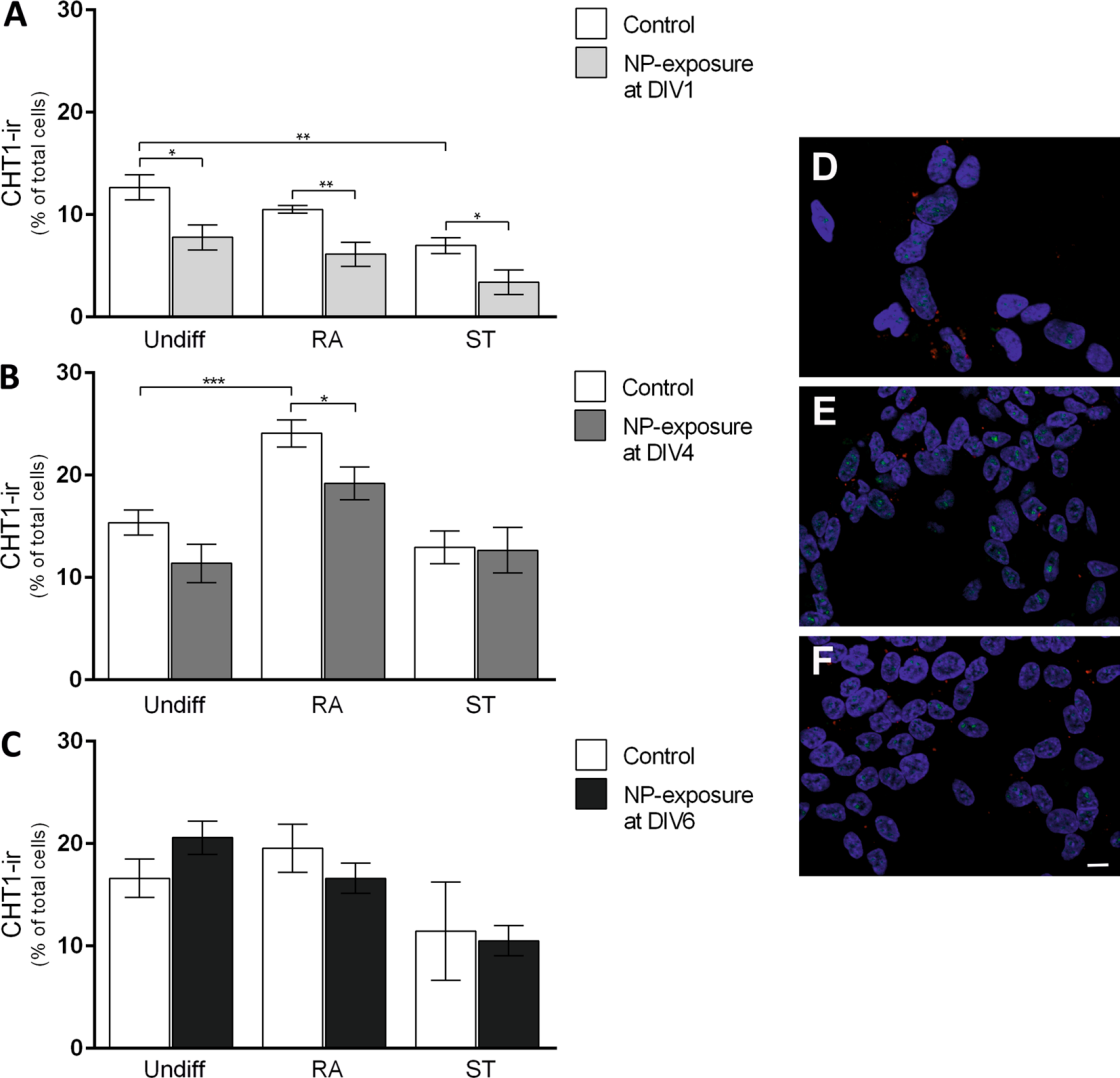
SH-SY5Y cells were exposed to SiPCL-NP at a concentration of (2.6 × 10^10^ NPs/ml) for 24 h and cells displaying a cholinergic phenotype were analyzed. NP-exposure was performed in undifferentiated cells (Undiff) or cells differentiated with retinoic acid (RA) or staurosporine (ST) at day in vitro (DIV) 1 (**A**), DIV4 (**B**) or DIV6 (**C**). At the end of the differentiation period (DIV7), high-affinity choline transporter 1-immunoreactive (CHT1-ir) cells were quantitatively assessed. Data represent the mean percentage (%) of CHT1-ir cells of the total number of cells. Error bars correspond to SEM. Significant differences between control- and SiPCL-NP-exposed-groups are labeled with asterisks (*p ≤ 0.05; **p ≤ 0.01; ***p ≤ 0.001). Representative immunofluorescence microscopy images of CHT1-ir cells (green) are depicted in **D**–**F** (**D** = Undiff NP DIV4, **E** = RA NP DIV4, **F** = ST NP DIV4). NPs are shown in red and cell nuclei in blue. Magnification ×630, scale bar = 10 μm

Semi-quantitative analyses showed an increased number of tyrosine hydroxylase (TH)-positive cells in ST-differentiated cells (15%) with the effect being similar to previously published findings [37, 38]. In contrast, RA did not increase TH-immunoreactive (-ir) cells when compared to undifferentiated cells (Fig. 3A–C). SiPCL-NP-exposure at DIV1 did not change the number of TH-ir cells in any of the tested conditions (undifferentiated cells, RA- and ST-treated cells) (Fig. 3A). A significant decrease in the number of TH-ir cells was observed when the SiPCL-NP-exposure was performed at DIV4 in RA-differentiated cells (Fig. 3B). A more prominent decrease in TH-ir cells was found in ST-differentiated cells after NP-application at DIV6 (Fig. 3C). TH-staining was barely detectable neither in undifferentiated cells (Fig. 3D) nor in RA-differentiated SH-SY5Y cells exposed to NPs at DIV4 (Fig. 3E). In contrast, TH was strongly expressed in ST-differentiated cells with the signal also being present in the neuronal processes (Fig. 3F). About 15% CHT1-immunoreactive (ir) cells were found in undifferentiated cells (Fig. 4A–C). ST-supplementation significantly reduced the number of CHT1-ir cells when compared to undifferentiated control cells (Fig. 4A). SiPCL-NP-exposure at DIV1 resulted in a significant decrease in the number of CHT1 cells at the end of differentiation regardless of the condition investigated, namely undifferentiated, RA- and ST-differentiated cells (Fig. 4A). A significantly lower number of CHT1-ir cells was found following NP-exposure at DIV4 in the RA-differentiated group (Fig. 4B). The number of CHT1-ir cells increased significantly when the cells were differentiated with RA compared to undifferentiated control cells (Fig. 4B). NP-treatment at DIV6 had no effect on the CHT1-ir cell number in any conditions tested (Fig. 4C). In the respective immunofluorescence images, the CHT1 signal was localized close to the nuclei and it showed a vesicular pattern (Fig. 4D–F). After SiPCL-NP exposure, high-affinity choline transporter 1 (CHT1)-ir cells were most prominent in RA-differentiated cells (Fig. 4E) when compared to undifferentiated cells (Fig. 4D) and ST-differentiated cells (Fig. 4F).

### Expression of phenotypical markers

**Fig. 5:**
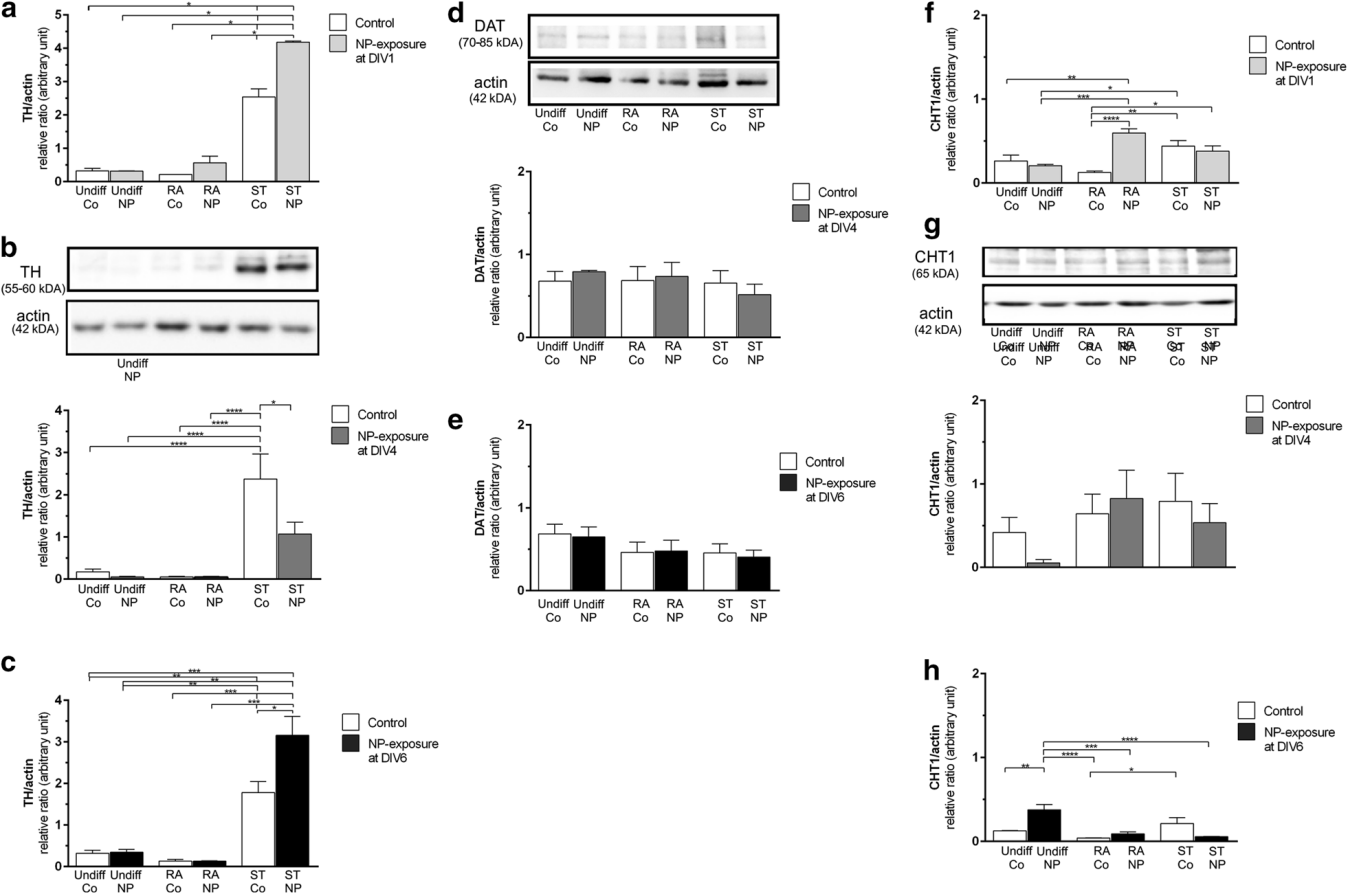
Analyses of tyrosine hydroxylase (TH) (**a**–**c**), dopamine active transporter (DAT) (**d**–**e**) and the high-affinity choline transporter 1 (CHT1) (**f**–**h**) were performed at day in vitro (DIV) 7 to evaluate the effect of SiPCL-NP-exposure for 24 h on DIV1 (**a**) and on DIV4 and DIV6 (**b**, **c**) on the phenotype of SH-SY5Y cells. Undifferentiated cells (Undiff) and cells differentiated with retinoic acid (RA) or staurosporine (ST) were investigated in parallel. Histograms depict the levels of TH, DAT and CHT1 in relation to the loading control (actin) given in arbitrary numbers. Representative Western blots are shown for DIV4 exposure above the respective histograms. Image J was used for the analysis. Error bars correspond to SEM. Significant differences between control- and NP-exposed-groups are labeled with asterisks (*p ≤ 0.05; **p ≤ 0.01; ***p ≤ 0.001; ****p ≤ 0.0001)

TH, the rate limiting enzyme in dopamine-synthesis, was significantly upregulated in ST-differentiated SH-SY5Y cells (10-fold) compared to undifferentiated and RA-treated SH-SY5Y cells (Fig. 5b). SiPCL-NP-exposure significantly decreased the expression of TH when differentiated with ST (Fig. 5b). SiPCL-NP-exposure at DIV1 (Fig. 5a) and DIV6 (Fig. 5c) resulted in a significant increase in TH-expression (15- to 20-fold) in ST-treated SH-SY5Y cells when compared to undifferentiated and RA-treated SH-SY5Y cells with or without NP-exposure at DIV1 (Fig. 5b). A 10- to 15-fold increase in TH-expression was found in control cells differentiated with ST at DIV6 (Fig. 5c). TH-expression was significantly higher after SiPCL-NP-exposure at DIV1 and DIV6 in ST-treated SH-SY5Y cells. The dopaminergic marker, dopamine active transporter (DAT), was not significantly altered regardless of the differentiation supplement and the time of NP-exposure (Fig. 5d, e). SiPCL-NP-exposure on DIV1 (Fig. 5f) resulted in a significant increase in CHT1-expression in RA-treated SH-SY5Y cells compared the respective RA-control, undifferentiated controls and undifferentiated-NP-exposed cells. ST-treated naïve and NP-exposed SH-SY5Y cells showed a significantly increased CHT1-expression when compared to RA-differentiated control cells and RA-differentiated NP-exposed cells (Fig. 5f). The expression of CHT1 was not significantly altered after differentiation with or without NPs at DIV4 or DIV6 when RA- or ST-differentiated cells were compared to undifferentiated cells (Fig. 5g, h). NP-exposure of undifferentiated SH-SY5Y cells at DIV6 resulted in a significant increase in CHT1-expression when compared to the undifferentiated controls. In both, RA-control and RA-exposed to NPs, the CHT1 expression was significantly lower than in undifferentiated NP-exposed cells. Similar findings were obtained for ST-treated cells exposed to NPs at DIV6. A significant increase of CHT1-expression was observed in RA-control compared to ST-control cells (Fig. 5h).

### Analysis of pathways involved in neurodifferentiation

**Fig. 6:**
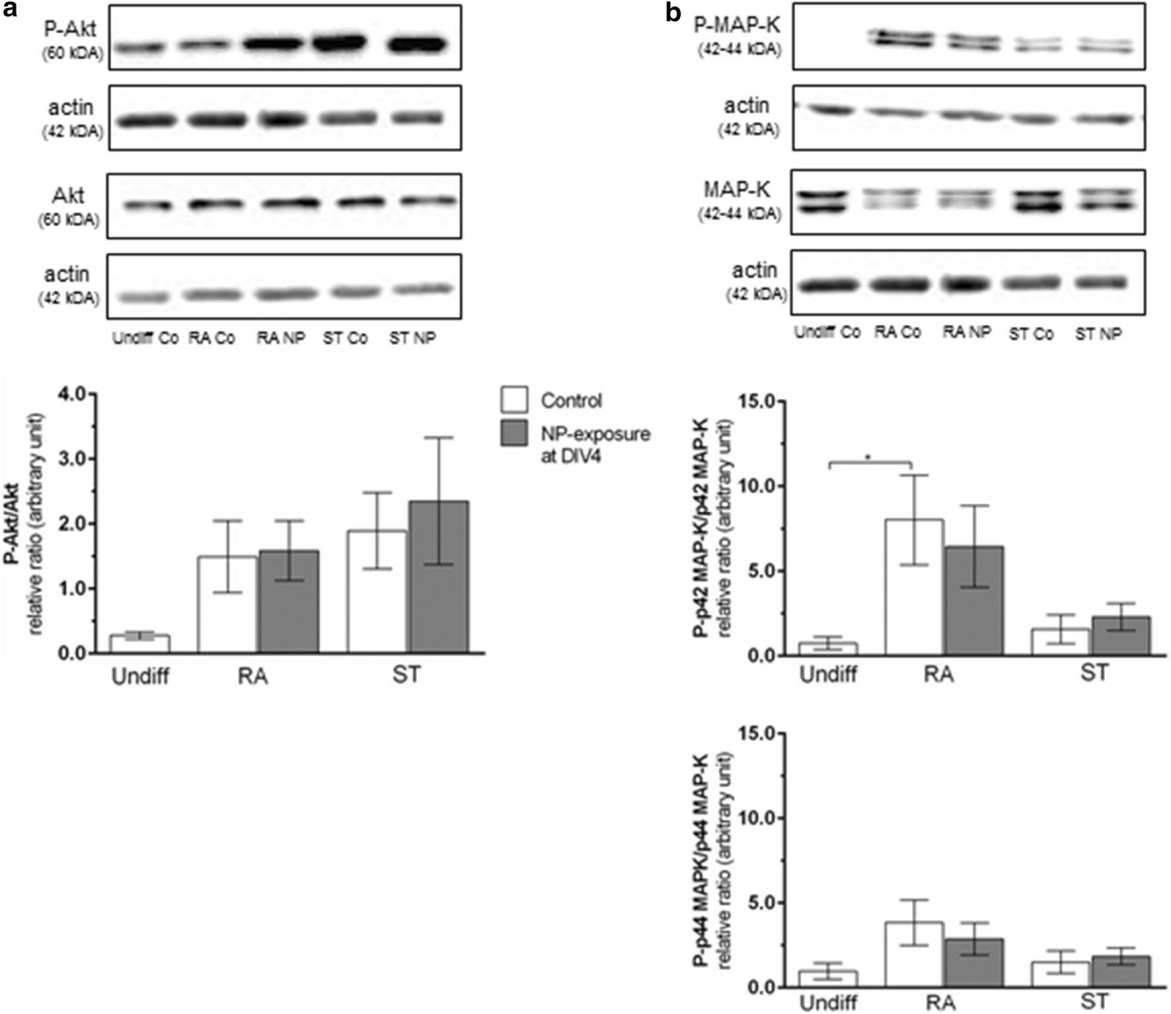
Analyses of differentiation markers [phosphatidylinositol-3-kinase (PI3-K/Akt) and of mitogen-activated-protein-kinase/extracellular-signal-regulated-kinase (MAP-K/ERK)] were performed at day in vitro (DIV) 7 to evaluate the effect of SiPCL-NP-exposure for 24 h on DIV4 in SH-SY5Y cells. Undifferentiated cells (Undiff) cells and cells differentiated with retinoic acid (RA) or staurosporine (ST) were assessed in parallel. Histograms (**a**, **b**) depict the ratio of phosphorylated Akt (P-Akt) (**a**) and phosphorylated MAP-K (P-p42/44 MAP-K) (**b**) to total Akt and p42/44 MAP-K, respectively. Actin was used as a loading control. Representative Western blots are shown above the histograms. Image J was used for the analysis. Error bars correspond to SEM. Significant differences between control- and NP-exposed-groups are labeled with asterisks (*p ≤ 0.05)

Signaling pathways involved in neuronal differentiation, namely Akt and MAP-K (Fig. 6a, b) were studied after SiPCL-NP-exposure. The expression of the active phosphorylated form of Akt was increased in RA- and ST-differentiated cells compared to undifferentiated controls but the effect was not statistically significant. SiPCL-NPs-exposure did not change the expression of Akt in SH-SY5Y cells (Fig. 6a). The active phosphorylated forms of MAP-K, namely p42 and p44, were upregulated in RA-differentiated controls (Fig. 6b). However, this upregulation was only statistically significant for p42 MAP-K (11.5-fold; p ≤ 0.05). SiPCL-NP-exposure for 24 h at DIV4 (2.6 × 10^10^ NPs/ml) did not affect the expression of the active phospho-p42 and phospho-p44 MAP-K.

**Fig. 7:**
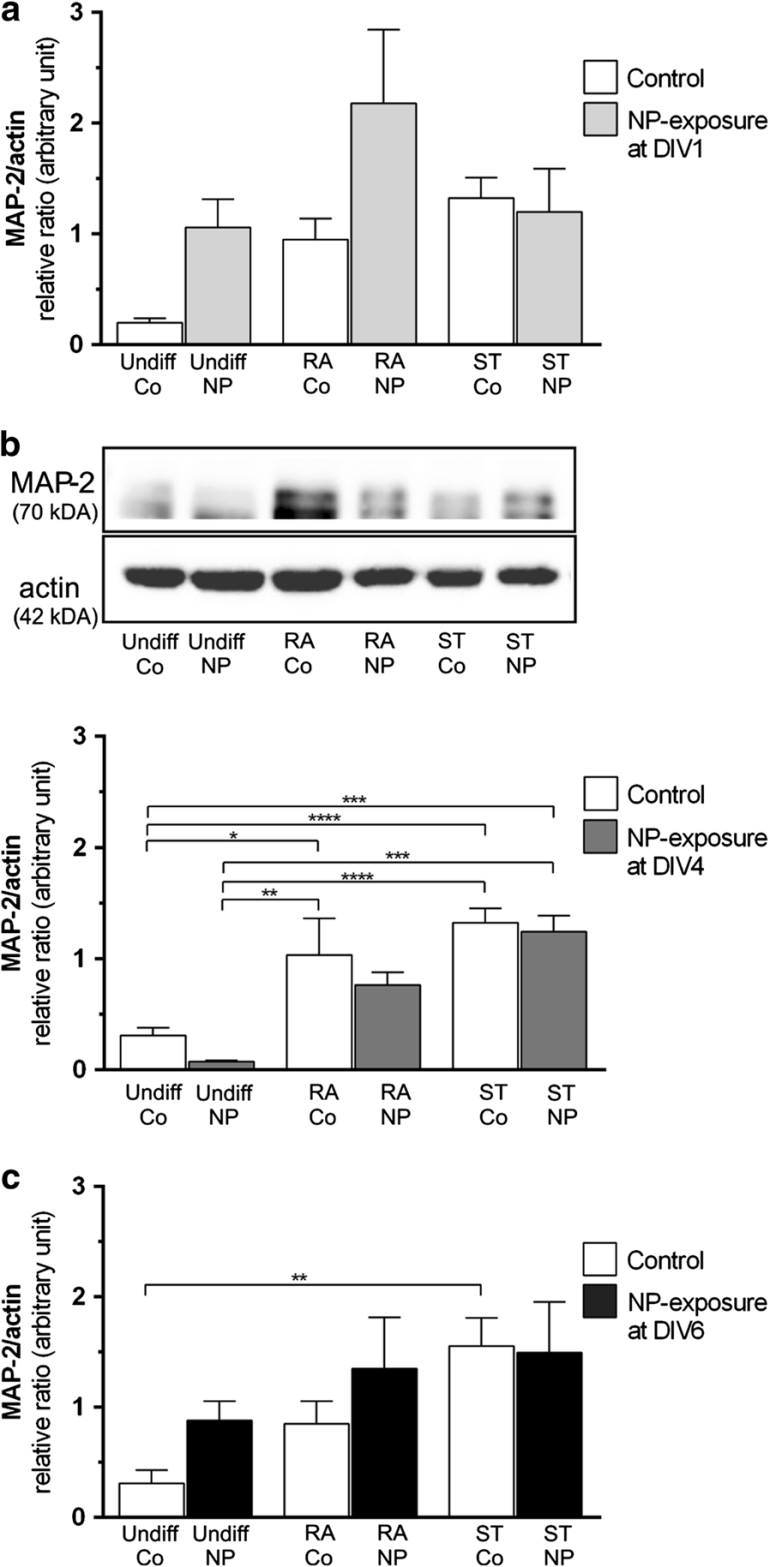
Analysis of the neuronal marker microtubule-associated protein 2 (MAP-2) was performed at day in vitro (DIV) 7 to evaluate the effect of SiPCL-NP-exposure for 24 h at DIV1 (**a**) and DIV4 (**b**) and DIV6 (**c**) on the state of differentiation in SH-SY5Y cells. Undifferentiated cells (Undiff) and cells differentiated with retinoic acid (RA) or staurosporine (ST) were examined in parallel. The histogram depicts MAP-2 levels in relation to actin (loading control). A representative Western blot for SiPCL-NP-exposure on DIV4 is shown above the respective histogram. Image J was used for the analysis. Error bars correspond to SEM. Significant differences between control- and NP-exposed-groups are labeled with asterisks (*p ≤ 0.05; **p ≤ 0.01; ***p ≤ 0.001; ****p ≤ 0.0001)

**Fig. 8:**
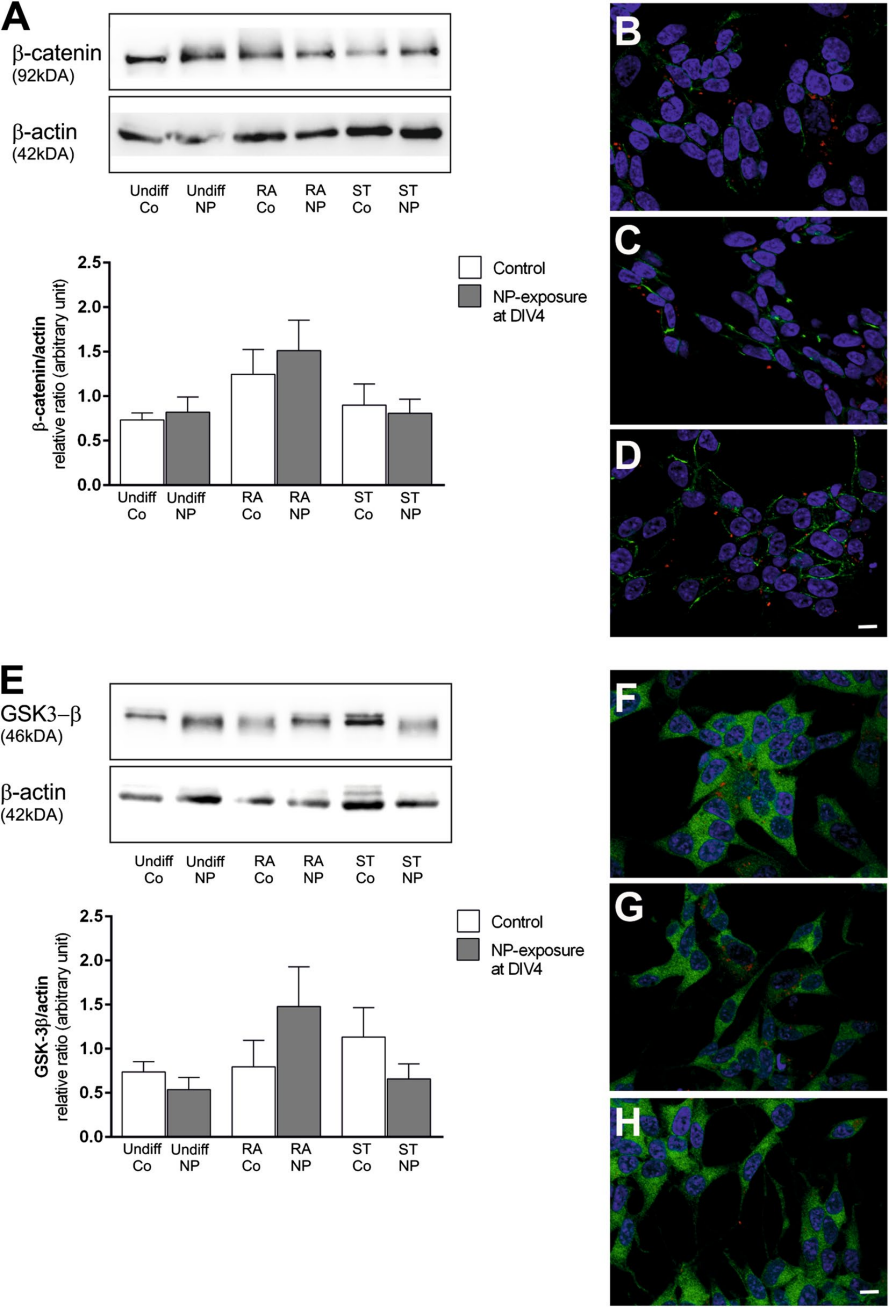
Analyses of the Wnt-pathway markers, β-catenin and glycogen synthase kinase-3 β (GSK-3β), were performed at day in vitro (DIV) 7 to evaluate the effect of SiPCL-NP-exposure for 24 h on DIV4 in SH-SY5Y cells. Undifferentiated cells (Undiff) and cells differentiated with retinoic acid (RA) or staurosporine (ST) were examined in parallel. Histograms depict β-catenin (**A**) and GSK-3β (**E**) levels in relation to the loading control (actin). Representative Western blots are shown above the histograms. Image J was used for the analysis. Error bars correspond to SEM. Representative immunofluorescence microscopy images of β-catenin-stained cells (green) are depicted in **B**–**D** (**B** = Undiff NP DIV4, **C** = RA NP DIV4, **D** = ST NP DIV4) and GSK-3β (green) in **F**–**H** (**F** = Undiff NP DIV4, **G** = RA NP DIV4, **H** = ST NP DIV4). NPs are shown in red and cell nuclei in blue. Magnification ×630, scale bar = 10 μm

Expression of the maturity marker, i.e. microtubule-associated protein 2 (MAP-2), was analyzed to estimate the differentiation state depending on the time of NP-exposure (DIV1 in Fig. 7a; DIV4 in Fig. 7b, DIV6 in Fig. 7c). Our data show an upregulation of MAP-2 in RA- and ST-differentiated cells compared to both undifferentiated control SH-SY5Y cells, with the effect being statistically significant for the RA- and ST-controls and ST-differentiated and NP-exposed cells at DIV4 (Fig. 7b). MAP-2-expression was not significantly altered by exposure to SiPCL-NPs at DIV4 for any differentiation supplement investigated (Fig. 7b). No significant differences in MAP-2 expression were observed when NP-exposure was performed at DIV1 (Fig. 7a) and DIV6 (Fig. 7c) for all groups investigated. However, a tendency towards increased MAP-2 levels was seen for RA- and ST-differentiated cells when compared to undifferentiated cells (Fig. 7a, c). An upregulation of activated (non-phosphorylated) β-catenin was obtained in RA-differentiated SH-SY5Y cells, but this effect was not statistically significant, and β-catenin levels were not affected by SiPCL-NP-exposure in undifferentiated, RA- and ST-treated SH-5YSY cells (Fig. 8A). β-catenin was found in or close to the cell membrane when analyzed by immunofluorescence microscopy. No apparent difference in the signal in undifferentiated and differentiated cells was obtained as illustrated in Fig. 8B–D. Furthermore, no co-localization of β-catenin and NPs was found (Fig. 8C, D). Differentiation did not alter glycogen synthase kinase-3β (GSK-3β) expression in SH-SY5Y cells. A slight but non-significant increase in GSK-3β-expression was found after NP-exposure in RA-differentiated cells, whereas the opposite was found after ST-differentiation (Fig. 8E). No variation was observed after NP-exposure in undifferentiated cells. GSK-3β was found in the cytoplasm with the signal being present in the entire cytoplasm (Fig. 8F–H). Microscopically, no obvious changes were noticed after SiPCL-NP-exposure (Fig. 8F–H).

## Discussion

RA-differentiated cells were most sensitive to SiPCL-NPs before differentiation (NP-exposure on DIV1). ST-differentiated SH-SY5Y cells were most impaired by NPs during differentiation (NP-exposure on DIV4). These findings might indicate that the NPs interfered with the mitochondrial activity of the cells at the beginning and during differentiation. SiO_2_-NPs sized 20 nm were reported to lead to an inhibition of mitochondrial respiratory chain complexes, the mitochondrial dehydrogenase activity and the mitochondrial membrane potential in rat liver cells [39]. In rats, SiNP-exposure (sized 80 nm) was also shown to induce mitochondrial dysfunction in cells of the corpus striatum, and electron microscopic studies provided evidence of neuronal mitochondrial dysfunction after SiNP-exposure in rats [8]. We recently demonstrated that SiPCL-NPs increased the mitochondrial membrane potential in SH-SY5Y cells under differentiation [14]. Reactive oxygen species were found in undifferentiated SH-SY5SY cells after exposure to PCL-NPs [12]. In agreement with our data, exposure to TiO_2_NPs (15 μg/ml) for > 24 h was reported to decrease the viability of SH-SY5Y cells by about 22% [40]. AgNP-exposure (16 and 32 μg/ml) for 24 h was also shown to reduce cell viability by 55% and 70% in undifferentiated SH-SY5Y [16]. In the neuron-like cell line PC12, a significant loss in cell viability was observed immediately after incubation with SiO_2_-NP for 24 h. While the decrease in cell viability was comparable with our data, the NP-concentration was about 10-times higher (200 μg/ml) [10] and the zeta potential and the size of the NPs differed from those in our study [10, 16, 40]. As demonstrated before, size and zeta potential affect the toxicity of NPs [41]. In contrast, previously published studies using SiPCL-NPs demonstrated no significant effect on cell viability in undifferentiated and RA-differentiated SH-SY5Y cells [12] and rat primary hippocampal cells [23] after NP-exposure for 24 h. While the NP-concentrations remained the same, the methylthiazolyldiphenyl-tetrazolium bromide (MTT)-based assay was used in this study, whereas Presto blue was used previously to analyze cell viability. There is evidence that mesoporous Si-NPs stimulated the MTT formazan exocytosis in astrocytes and the MTT reduction test was shown to rather overestimate the cytotoxicity [42]. The tetrazolium-based MTT assay has long been regarded as the gold standard for cytotoxicity assays as it is highly sensitive, but it has been demonstrated to be less accurate in detecting changes in cell numbers [43]. Moreover, MTT-reduction was shown to be significantly affected by metabolic and energy perturbations. As metabolic changes are known to occur during differentiation and NPs were shown to affect these changes [14, 44], an overestimation of the cytotoxicity is likely.

In accordance with this finding, previous studies using transmission electron microscopy (TEM) showed SiPCL-NPs in the cytoplasm and intracellular vacuoles in RA-differentiated SH-SY5Y cells [12]. Quantification of the NP-uptake revealed that 30% of the neurons had taken up SiPCL-NPs [23]. An uptake of Si-NPs has also been demonstrated in primary rat microglia using fluorescence microscopy and TEM [15]. However, it needs to be pointed out that the Si-NPs used in the present study where coated with PCL on the outside. Hence, the cells did not get in touch with the silica core of the NPs, which may also explain the different findings. Si-NPs have also been shown to infiltrate neuronal cell bodies and axonal projections of *Drosophila melanogaster* neurons and again, they have been found in the cytoplasm but not in the nucleus [11]. While astrocytes and neuronal stem cells were reported to take up Si-NPs, no uptake was seen in neurons [45]. Different coatings and NP-size as well as the cell type are known to affect the uptake of NPs [46–48]. Even though SiPCL-NPs were taken up by SH-SY5Y cells, the cell morphology was not obviously altered in our experiments. Consistent with this finding, Ducray et al. [14] demonstrated that SiPCL-NPs did not cause clear morphological alterations in SH-SY5Y cells. In the present study, the morphology of cholinergic and dopaminergic neurons was not affected by the NP-exposure.

In agreement with other studies, CHT1-ir cells were primarily seen in undifferentiated and RA-differentiated SH-SY5Y cells [49–51]. SiPCL-NPs decreased the number of cholinergic cells. Unlike the dopaminergic phenotype, this effect was found when the NPs were incubated before the differentiation, which might indicate that the cholinergic cells are more vulnerable in the beginning of the differentiation process, whereas the dopaminergic cells are more vulnerable towards the end.

Although SiPCL-NP-treatment in the middle of differentiation did not result in a decrease of TH-ir cells in ST-differentiated cells, protein quantification revealed a decrease in the number of TH-positive cells after SiPCL-NPs-exposure. Similarly, a significant decrease in dopaminergic cells was also found when the SiPLC-NPs were added to the cells at the end of differentiation (NP-exposure on DIV6), indicating that dopaminergic cells at the later state of differentiation might be more vulnerable to NP-exposure. However, the number of cells displaying the dopaminergic phenotype increased with time. Therefore, a difference in dopaminergic cells might be difficult to detect in the beginning and in the middle of the differentiation process. Consistent with these findings, the expression of TH was downregulated concentration-dependently in PC12 cells exposed to Si-NPs (25–200 μg/ml) for 24 h [10]. Corroborating our findings, a decrease in TH-expression was demonstrated after Si-NP-exposure in other experimental models, namely in the substantia nigra of zebrafish [52], in olfactory bulb neurons of neonatal rats after treatment with nano-sized TiO_2_ [53] and in the midbrain of rats after intracerebral injection of manganese-NPs [54]. On the other hand, an alteration of the dopaminergic marker DAT was not observed after exposure to SiPCL-NP. In agreement with this result, Si-NPs did not alter the expression of DAT in PC12 cells [10].

Dayem et al. [22] also showed an increase in levels of phosphorylated-Akt and -ERK, proteins that have been demonstrated to play an important role in neurite elongation (PI3-K/Akt), in neuronal survival and in synaptic plasticity (MAP-K/ERK) [27, 55, 56]. Primary astrocytes treated with ZnO-NPs were demonstrated to increase ERK- and MAP-K-phosphorylation (p38) [24]. In the present study, key markers of signalling pathways involved in differentiation (pAKT and MAPK as well as GSK-3 and beta-catenin) were studied after SiPCL-NP-exposure at DIV4 because we hypothesized that significant changes in the respective phenotype may be accompanied by alterations of these markers. SiPCL-NPs did not change the expression of Akt and MAP-K regardless of the differentiation supplement used. Ducray et al. [14] also did not observe an alteration in the expression of phosphorylated-Akt after SiPCL-exposure in RA-differentiated SH-SY5Y cells. A concentration-dependent decrease has also been reported for the active and phosphorylated forms of Akt and PI3K after SiO_2_-NPs exposure in PC12 cells [28]. ZnO_2_-NPs with a size of 30 nm and 45 nm and Ag-NPs were reported to activate the PI3K/Akt- and MAP-K/ERK-pathway in primary astrocytes and SH-SY5Y cells, respectively [22, 24]. On the contrary, SiO_2_-NPs, sized 25 nm and with a negative zeta potential (− 8.8 mV), exhibited adverse effects on the differentiation of PC12 cells [28]. These results demonstrate again that physicochemical properties of NPs affect toxicity and uptake [47]. In contrast, MAP-2 was shown to be slightly upregulated after SiPCL-NP-treatment in SH-SY5Y cells [14]. Ag-NP-exposure was demonstrated to significantly enhance the amount of mature neurons [22], which might be due to the different material of the NPs.

Markers involved in neuronal differentiation and neurodegeneration, namely GSK-3β and β-catenin were not significantly altered by SiPCL-NPs in the present study regardless of the methods used. However, the NP-treatment induced a slight increase of GSK-3β in RA-differentiated cells, which might result in a subsequent depletion of β-catenin. In contrast, the non-significant downregulation of GSK-3β in ST-differentiated cells is likely to prevent degradation of β-catenin and prevent neurodegeneration. As opposed to our results, TiO_2_-NP-exposure was shown to increase the expression of GSK-3β and to downregulate β-catenin levels in primary rat hippocampal neurons [33]. It is important to consider that an upregulation of GSK-3β is linked to degradation of β-catenin and alterations in this signaling cascade have been reported to be related to the pathogenesis of neurodegenerative diseases [57, 58].

## Conclusions

The findings obtained from SiPCL-NP-exposure in SH-SY5Y cells undergoing differentiation indicate an impairment of the resulting dopaminergic and cholinergic phenotype with the effect depending on the time of the NP-application during neuronal differentiation. The decrease in the number of cells representing the dopaminergic or the cholinergic phenotype may implicate neuronal dysfunction, but the data do not provide evidence that pathways relevant for differentiation and related to neurodegeneration are impaired.

## Methods

### Cell culture

The human neuroblastoma cell line SH-SY5Y was purchased from ATCC (Manassas, VA, USA) and cultured as reported previously [14]. For the experiments, SH-SY5Y cells of passage 5 to 21 at a confluency of 65–85% were used.

The differentiation was carried out according to a previously published protocol with minor changes [14]. Briefly, the cells were seeded and kept in an undifferentiated state for 24 h. For differentiation, plates and flasks were coated with poly-dl-lysine hydrobromide (0.1 mg/ml) (96-well-plates, µClear^®^ CELLSTAR^®^, Greiner Bio-One, AUT) or polyornithine 0.01%/laminin [6 μg/ml] (Sigma-Aldrich, Switzerland) on glass coverslips (VWR International, USA) (24-well-plates, Techno Plastic Products, Trasadingen, Switzerland). The differentiation procedure started at DIV 1. The protocol consisted of two phases of 3 days each. In phase one on DIV1, the differentiation was induced by a 5% reduction of FBS in the medium and it was further reduced to 1% in phase two on DIV4. In both phases, the medium was supplemented with either RA at a concentration of 10 μM (Sigma-Aldrich, Switzerland) or ST [5 nM] (Sigma-Aldrich, Switzerland). Undifferentiated controls were kept in the same medium for the entire culture period and medium changes were done at the same time point as for the differentiated cells.

### Nanoparticle exposure

SiPCL-NP synthesis, chemical and physical characteristics were reported previously [6, 12, 14]. Briefly, the SiPCL-NPs had an average size of 80 nm and a negative Zeta potential of − 25.4 mV. Rhodamine-labeled SiPCL-NPs were used for identification using fluorescence microscopy.

The NP-stock-solution was prepared at a concentration of 2.6 × 10^11^ NPs/ml in Dulbecco’s phosphate-buffered saline (Life Technologies, UK) with 0.25% dimethyl sulfoxide (DMSO) (Sigma-Aldrich, Switzerland). Shortly before exposure to the cells, the NP-stock-solution was sonicated three times for 4 min with 4 min cooling steps between each sonication in order to prevent inhomogeneous distribution in the suspension as previously described [14]. NP-exposure was performed at a final concentration of 2.6 × 10^10^ NPs/ml in 1% FBS culture medium for 24 h either at the beginning, in the middle or at the end of the differentiation, i.e. on DIV1, DIV4 and DIV6, respectively. Cells devoid of SiPCL-NPs were used as controls throughout each in vitro assay.

### Cell viability

Cell viability was measured spectrophotometrically using the methylthiazolyldiphenyl-tetrazolium bromide (MTT) colorimetric assay (Sigma-Aldrich, Switzerland) in control cells and the three groups exposed to NPs, namely cells exposed on DIV1, DIV4 and DIV6. SH-SY5Y cells were seeded in 96-well MICROTEST tissue culture plates (Falcon BD, USA) at a cell density of 8.4 × 10^3^ cells per well. On DIV7, three independent experiments were performed for each group. Briefly, MTT was added to the cells at a final concentration of 0.5 mg/ml. After incubation for four h at 37 °C in 5% CO_2_, the precipitated formazan was dissolved in DMSO. Changes in absorbance were measured with the Synergy H1 microplate reader (BioTek, USA) at a wavelength of 540 nm.

### Phenotypical characterization

For phenotypical characterization, SH-SY5Y cells were seeded in 24-well tissue culture plates at a cell density of 2.8 × 10^4^ cells per well. SiPCL-NP-exposure was performed on DIV4. At DIV7, the cells were sequentially fixed in 2% and in 4% paraformaldehyde (Sigma-Aldrich, Switzerland) for 10 min each. Subsequently, the fixed cells were blocked for two h in 0.4% Triton-X (Sigma-Aldrich, Switzerland) in phosphate-buffered saline (PBS) (Life Technologies, UK) with 10% horse serum (Thermo Fisher Scientific, Switzerland). The primary antibodies (see Table 1) were diluted in a stock solution (0.4% Triton-X PBS with 2.5% horse serum) and incubated with the cells overnight at 4 °C. The following day, the secondary antibodies (see Table 2) were diluted in the stock solution (see above). Hoechst 33,342 nucleic acid stain at a dilution of 1:10,000 (Thermo Fisher Scientific, Switzerland) was added and the cells were incubated for 2 h at room temperature in the dark. After mounting the cover slips on slides with Glycergel Mounting Medium (Agilent Technologies, USA), immunofluorescence pictures were obtained with a Zeiss Axio Imager Z1 coupled with an Apotome 1 (Carl Zeiss Vision Swiss AG, Feldbach, Switzerland).

**Table 1:** Primary antibodies

Primary antibody	Host	Dilution		Company
		IF	WB	
phospho-Akt	Rabbit	–	1:1000	Cell signaling
Akt	Rabbit	–	1:1000	Cell signaling
GSK-3β	Rabbit	1:400	1:1000	Cell signaling
TH	Rabbit	1:500	1:1000	Cell signaling
β-catenin	Rabbit	1:400	1:500	Cell signaling
phospho-p44/42 MAP-K	Mouse	–	1:1000	Cell signaling
p44/42 MAP-K	Mouse	–	1:1000	Cell signaling
CHT1	Mouse	1:250	1:500	Santa Cruz
β-actin	Mouse	–	1:20,000	Sigma-Aldrich
MAP-2	Mouse	–	1:500	Sigma-Aldrich
β-3-tubulin	Mouse	1:500	–	Sigma-Aldrich
α/β-synuclein	Mouse	1:250	–	Santa Cruz
DAT	Rat	–	1:1000	Millipore

*CHT1* choline transporter 1, *DAT* dopamine active transporter, *GSK-3* β glycogen synthase kinase 3 β, *IF* immunofluorescence, *MAP-2* microtubule associated protein 2, *MAP-K* mitogen activated protein kinase, *TH* tyrosine hydroxylase, *WB* western blot

**Table 2:** Antibodies

Secondary antibody	Dilution		Use with corresponding primary for detection of	Company
	IF	WB		
dk anti-rabbit IgG HRP	–	1:5000	phospho-Akt; Akt; TH	Novex
	–	1:10,000	GSK3-b; β-catenin	
dk anti-rabbit IgG AF 488 or 647 nm	1:200	–	GSK3-b; β-catenin; TH	Invitrogen
dk anti-mouse IgG HRP	–	1:5000	phospho-p44/42 MAP-K; p44/42 MAP-K; MAP-2	Novex
	–	1:50,000	β-actin	
dk anti-mouse IgG AF 488 nm	1:200	–	CHT1; β-3-tubulin; α/β-synuclein	Invitrogen
dk anti-rat IgG HRP	–	1:2000	DAT	Novex

*AF* Alexa Fluor, *dk* donkey, *HRP* horseradish peroxidase, *IF* immunofluorescence, *WB* Western blot

### Quantification of cellular phenotype

Quantification of dopaminergic and cholinergic phenotypes was performed using high-content analysis in control cells and cells exposed to SiPCL-NPs on DIV1, DIV4 and DIV6. The SH-SY5Y cells were seeded in a 96-well microplate at a cell density of 8.4 × 10^3^ cells per well. On DIV7, the cells were fixed and blocked as described above. The primary antibodies against TH and CHT1 (see Table 1) were diluted in a stock solution consisting of 0.4% Triton-X PBS with 2.5% horse serum, and the incubations with the cells were performed overnight at 4 °C.

As described above, the secondary antibodies (Table 2) were applied together with Hoechst 33342 nucleic acid stain on the following day. For the quantitative analysis of TH- ir and CHT1-ir cells, high content analysis of the SH-SY5Y cells was performed with an INCell Analyzer 2000 (INCA) (General Electric Healthcare Europe GmbH, Glattbrugg, Switzerland).

Images were taken from three wells per group and 35 randomly assigned areas for each well. The total number of cells, evaluated by the number of nuclei, and TH- or CHT1-ir cells were determined using the INCell Developer Toolbox 1.9.2 (General Electric Healthcare Europe GmbH, Glattbrugg, Switzerland).

### Analysis of markers involved in neuronal differentiation

Markers of pathways involved in neuronal differentiation, namely Akt, MAP-K, GSK-3β, β-catenin, MAP-2, TH, DAT, and CHT1, were studied after SiPCL-NP-exposure at DIV1, 4 and 6. SH-SY5Y cells were seeded in a 75 cm^2^ tissue culture flask (Techno Plastic Products, Trasadingen, Switzerland) at a cell density of 1.3–1.4 × 10^6^ cells per flask before differentiation and exposure to SiPCL-NPs (DIV4). On DIV7, total cellular protein was extracted using cold protein lysis buffer containing phosphatase (Sigma-Aldrich, Switzerland) and protease (Thermo Fisher Scientific, Switzerland) cocktail inhibitors. Protein (10 μg) from each sample was loaded and separated on 10% SDS-Page gels. Thereafter, the transfer from the gel to a polyvinylidene difluoride membrane (Bio-Rad Laboratories, USA) or a nitrocellulose membrane (GE Healthcare Life science, UK) was performed. Blocking of the membranes was accomplished with the stock solution containing 0.2% TWEEN 20 (Sigma-Aldrich, Switzerland) in PBS with 5% milk powder for two h. Incubations with the primary antibodies (see Table 1) were performed overnight at 4 °C in the stock solution. Finally, the membranes were incubated for 2 h in the stock solution with the corresponding horseradish peroxidase (HRP) conjugated secondary antibody (see Table 2). The bands were detected using chemiluminescent substrate (Advansta Western Bright Sirius Chemiluminescent, Witec, Switzerland) and a CCD-LAS3000 camera (Fujifilm, Japan). The blots were quantified with ImageJ analysis (National Institutes of Health, USA) using arbitrary units to determine the ratio between the band-intensity of the respective marker and the corresponding band of actin.

### Statistical analysis

For each experiment, at least three independent experiments were performed. Statistical analysis was carried out with GraphPad Prism 6 (GraphPad Software Inc., USA). Student’s unpaired t-test was used to analyze cell viability, TH- and CHT1-ir cells in SiPCL-NP-exposed and control cultures. A one-way ANOVA test followed by Tukey’s multiple comparison test was used for the analysis of differentiation and neuronal markers. The results are presented by mean ± standard error of the mean (SEM). P-values of ≤ 0.05 were considered as statistically significant.

## Abbreviations

AgNPs: silver nanoparticles
BSA: bovine serum albumin
CHT1: high-affinity choline transporter 1
CNS: central nervous system
DAT: dopamine active transporter
DIV: day in vitro
DMSO: dimethyl sulfoxide
ERK: extracellular-signal-regulated-kinase
FBS: fetal bovine serum
GSK-3β: glycogen synthase kinase-3 β
HRP: horseradish peroxidase
ir: immunoreactive
MAP-2: microtubule-associated protein 2
MAP-K: mitogen-activated-protein-kinase
MTT: methylthiazolyldiphenyl-tetrazolium bromide
NPs: nanoparticles
PBS: phosphate-buffered saline
PI3K: phosphatidylinositol-3-kinase
RA: retinoic acid
Si-NPs: silica nanoparticles
SiPCL-NPs: silica-ε-polycaprolactone-nanoparticles
ST: staurosporine
TEM: transmission electron microscopy
TH: tyrosine hydroxylase
TiO_2_-NPs: titanium dioxide NPs
ZnONPs: zinc oxide NPs
